# The Reliability and Validity of the Greek Version of the Scale for the Identification of Symptoms and Risks of Child Abuse and Neglect (SISRCAN) for Midwives

**DOI:** 10.7759/cureus.90790

**Published:** 2025-08-23

**Authors:** Eleni Theodoridou, Athanasios Sachlas, Alexandra Soldatou, Victoria Vivilaki, Angeliki Antonakou

**Affiliations:** 1 Department of Midwifery, International Hellenic University, Thessaloniki, GRC; 2 Department of Computer Science and Biomedical Informatics, University of Thessaly, Lamia, GRC; 3 2nd Department of Pediatrics, National and Kapodistrian University of Athens, “P&amp;A Kyriakou” Children’s Hospital, Athens, GRC; 4 Department of Midwifery, University of West Attica, Athens, GRC; 5 Department of Midwifery, School of Health Sciences, International Hellenic University, Thessaloniki, GRC

**Keywords:** child maltreatment, healthcare professionals, knowledge assessment, questionnaire, secondary prevention

## Abstract

Background

Child abuse and neglect (CAN) are global issues with profound implications for child health and safety. Midwives play a critical role in early detection and prevention. However, there is limited evidence concerning Greek midwives’ knowledge of CAN. This study aimed to translate, culturally adapt, and validate the Greek version of the Scale for the Identification of Symptoms and Risks of Child Abuse and Neglect (SISRCAN) and to assess midwives’ knowledge of CAN.

Methods

The SISRCAN questionnaire, originally developed in Turkey, was translated and validated in accordance with World Health Organization (WHO) guidelines. A pilot study (n=70) and a nationwide survey (n=493) were conducted among registered Greek midwives. Internal consistency, test-retest reliability, and construct validity were assessed using Cronbach’s alpha, intraclass correlation coefficients (ICC), and Pearson’s correlation. Descriptive statistics were used to analyze participants’ demographics and SISRCAN scores.

Results

The Greek version of SISRCAN demonstrated high internal consistency (α=0.889-0.929) and strong test-retest reliability (ICC=0.947; p<0.001). The mean total SISRCAN score was 239.23 (standard deviation {SD}=23.60), indicating moderate knowledge. Most participants (93.7%) had not received formal training on CAN, and 72% rated their relevant training inadequate. The lowest scores were found in the subscale assessing the knowledge of child characteristics associated with increased CAN risk, while the subscale “symptoms of neglect on the child” received the highest mean score among all subscales.

Conclusions

The validated Greek SISRCAN is a reliable instrument for assessing midwives’ knowledge of CAN. Results highlight substantial gaps in formal education and training on child abuse and neglect among Greek midwives. Integrating structured CAN education into midwifery curricula and ongoing professional development is essential for improving early recognition and intervention.

## Introduction

Violence against children remains a persistent, global public health and human rights crisis. It is complex, multidimensional, and deeply rooted in societal, cultural, and systemic factors. The World Health Organization (WHO) defines violence against children as “the intentional use of physical force or power, actual or threatened, against children under 18 years of age that either results in or has a high likelihood of resulting in injury, death, psychological harm, maldevelopment, or deprivation” [1]. These acts of violence can be committed by adults, whether caregivers, relatives, or strangers, or even by peers.

Globally, the burden of child abuse and neglect (CAN) is staggering. According to the WHO, nearly 400 million children under the age of five, approximately six in 10, experience regular physical or psychological violence from parents or caregivers [2]. The Council of Europe estimates that one in five children has experienced some form of violence, such as rape, exploitation, or bullying, where in 70%-85% of cases, the perpetrator was known to the victim [3]. These early adverse experiences have lifelong implications for health, development, and social integration, contributing to the intergenerational cycle of abuse and inequality.

In Greece, the lack of a centralized registry or national surveillance system for child maltreatment significantly hampers the accurate estimation of its prevalence. Existing data, drawn primarily from isolated epidemiological studies or child protection agencies, suggest that the issue is underreported and often inadequately addressed. This gap in national monitoring underscores the urgent need for proactive identification tools and frontline intervention strategies, particularly within maternal and child healthcare settings.

Midwives, as primary healthcare providers who support families from the prenatal period onward, are strategically positioned to identify early warning signs and risk factors of child abuse and neglect. Their role is especially critical in the perinatal period, when signs of familial stress, socioeconomic vulnerability, or potential neglect may begin to emerge. However, there is limited research examining midwives’ knowledge and competencies in this area, particularly in the Greek context.

To our knowledge, no validated instrument currently exists in the Greek language to assess midwives’ ability to recognize early symptoms and risks of child abuse and neglect (CAN). The “Scale for the Identification of Symptoms and Risks of Child Abuse and Neglect” (SISRCAN), developed by Prof. Uysal, has been widely used in Turkey for nearly two decades to support training and early intervention among healthcare professionals [4]. Validating a Greek version of this instrument will help standardize assessment practices and strengthen midwifery education on child protection.

This effort supports several key United Nations Sustainable Development Goals (SDGs). It advances SDG 3 by promoting the early detection and prevention of child abuse, essential for reducing child morbidity and mortality. It contributes to SDG 4 through targeted training for midwives in child protection. SDGs 5 and 10 are addressed by recognizing the disproportionate impact of abuse on girls and marginalized children. Finally, it aligns with SDG 16 by reinforcing systems that uphold children’s safety and rights.

This study aims to translate and validate the Greek version of the SISRCAN instrument, evaluate its reliability for use by midwives, and measure their knowledge of early symptoms and risk factors associated with child abuse and neglect. By doing so, it seeks to empower midwives with evidence-based tools for early identification and timely intervention, thus contributing to national and global efforts to prevent violence against children.

## Materials and methods

SISRCAN was developed by Prof. Uysal to determine the knowledge levels of nurses and midwives in diagnosing the symptoms and risks of CAN [4]. It consists of 67 statements, divided in six subscales, including the following: physical symptoms of abuse on the child (19 items), child’s behavioral symptoms of abuse (15 items), symptoms of neglect on the child (seven items), features of the parents having tendency to abuse and neglect (12 items), characteristics of children having tendency of being abused and neglected (six items), and familial characteristics regarding child abuse and neglect (12 items). Every item is rated on a five-point Likert scale ranging from “very correct” (five points), “quite correct” (four points), “undecided” (three points), “not quite correct” (two points), and “not correct at all” (one point). Items 3, 5, 8, 10, 12, 14, 16, 27, 28, 30, 32, 34, 41, 42, 46, 49, 54, 56, 59, 61, and 63 of the scale are reverse-scored, and the total score ranges from 67 to 335. A score below 3 indicates an incorrect answer, and a score close to 5 indicates a correct answer.

Demographic and background details encompass both personal and professional attributes, including age, gender, marital status, the length of professional experience, current role, and any direct or indirect experiences with CAN. These may involve having been a victim, knowing someone who was a victim, or having previously identified and reported suspected cases of CAN.

SISRCAN showed evidence of construct validity and reliability, and the final Cronbach’s alpha was 0.924 with subscales ranging from 0.596 to 0.890 [4]. SISRCAN is widely used in surveys conducted in Turkey [5-18].

Several factors made SISRCAN an ideal choice for assessing CAN knowledge among Greek nurses and midwives. The tool demonstrates strong psychometric properties, including high reliability, with an overall Cronbach’s α of 0.924 across its six domains of abuse and neglect recognition. Its construct validity has been previously established in a large Turkish sample, ensuring each subscale effectively captures distinct aspects of symptom recognition or risk profiling. Moreover, SISRCAN was originally designed for midwives and nurses, making it directly relevant to the clinical responsibilities, observations, and decision-making processes of Greek midwives. With 67 items covering a broad spectrum, physical signs, behavioral indicators, parental risk factors, child vulnerability markers, and family characteristics, the tool comprehensively addresses the child-safeguarding concerns Greek professionals encounter.

Cultural and practical factors further support SISRCAN’s use in the Greek context. The cultural and healthcare system similarities between Greece and Turkey, such as shared family structures, social attitudes toward child welfare, and comparable midwifery training frameworks, enhance the tool’s interpretability without requiring extensive adaptation. These parallels ensure that Greek users readily understand clinical vignettes and symptom descriptions. Additionally, SISRCAN offers practical advantages for cross-national comparison, facilitating benchmarking with Turkish data to identify common knowledge gaps or successful interventions. Since only minor linguistic and contextual adjustments were needed, the tool retained its original validity while saving time and resources. Collectively, these attributes affirm SISRCAN as the optimal instrument for evaluating CAN knowledge among Greek midwives and nurses.

We followed the World Health Organization’s recommended procedures for instrument translation and adaptation after receiving the scale’s developer, Prof. Uysal’s, permission to use the SISRCAN questionnaire. Specifically, we conducted independent forward translations by two translators, reconciled differences to create a single version, performed back-translation, carried out an expert committee review, conducted pretesting and cognitive interviews, finalized the instrument, and completed psychometric validation [19]. A panel of five Greek professionals, comprising three midwives, a pediatrician, and a lawyer specializing in child abuse, assessed the SISCRAN tool for content validity. Content validity indices were determined based on the level of agreement among the experts.

Ethical approval for the study was secured from the Research Ethics Committee of the International Hellenic University (approval number: 20/02.02.2023, issued on February 2, 2023) before the research commenced. This study forms part of the principal investigator’s (ET) PhD project.

To be eligible for inclusion in the study, participants were required to be licensed midwives who had completed their education, were actively practicing in Greece, and were proficient in the Greek language. All midwives who met these criteria and voluntarily agreed to participate were deemed eligible. Participants were informed of their right to withdraw from the study at any time without any negative consequences. Each questionnaire was accompanied by an information leaflet and a consent form, which included the contact details of both the primary researcher and the supervising professor, enabling participants to seek clarification if needed. The completion and return of the questionnaire were considered to imply informed consent.

To ensure confidentiality, no personal identifiers were obtained. All data were collected, stored, and analyzed in compliance with applicable data protection regulations, with strict measures in place to safeguard participant anonymity and confidentiality throughout the research process. There were no exclusion criteria other than the late submission of questionnaires. Approval for the study protocol was obtained from the scientific committees of two private and three public maternity hospitals in Athens, granting access to these sites for data collection. To assess test-retest reliability, a sample of 70 midwives completed the questionnaire twice, with a one-week interval between submissions.

Based on Viechtbauer et al.’s formula for determining sample size in pilot studies, a minimum of 59 participants is needed to detect issues with a 5% prevalence at a 95% confidence level [20]. For questionnaire validation, a convenience sample of 70 midwives was selected. For the preliminary research, the procedure of data collection took place in Athens, Greece, between February and June 2023. The sample used in the main study was adequate to estimate a mean with 90% statistical power and a precision of two at a 5% significance level. Simple random sampling was employed for sample collection. To ensure a representative sample, participants in the main study were recruited from Athens, as well as multiple other regions throughout Greece, from October 2023 to June 2024.

Statistical analysis

Absolute and relative frequencies were used to summarize categorical variables, while measures of central tendency and variability were applied to continuous variables. The internal consistency of the questionnaire was evaluated using Cronbach’s alpha. Pearson’s correlation coefficient was employed to assess the instrument’s repeatability, whereas test-retest reliability was examined through the intraclass correlation coefficient (ICC) and the paired samples t-test. All statistical analyses were performed using IBM SPSS Statistics (version 29.0, IBM Corp., Armonk, NY) for Windows, with the significance level set at 5% for all tests.

## Results

Preliminary validation study

We conducted a validation study, with a sample of 70 midwives, to assess the reliability and construct validity of the questionnaire before its use in the main research. Cronbach’s α coefficient, after reversing the questions with reverse meaning, was found to be equal to 0.929 and 0.923 during the test and retest, respectively. Both values are considered particularly high. Cronbach’s α coefficient, for the six subdimensions of the SISRCAN scale, for both the test and retest of the questionnaire, is presented in Table 1. Except for the subdimension “characteristics of children prone to abuse and neglect,” which had particularly low internal consistency, the remaining subdimensions presented good internal consistency. One contributing factor to the low reliability scores may be the high proportion of participants who provided similar responses across items within this subscale. This uniformity reduced the variability needed to effectively evaluate reliability, which in turn may have led to an artificial reduction in internal consistency estimates [21].

**Table 1:** Cronbach’s alpha coefficient of subscales of SISRCAN for test and retest (n=70) Cronbach’s alpha coefficients for each of the six subscales of the SISRCAN instrument during the initial test and retest phases CAN, child abuse and neglect; SISRCAN, Scale for the Identification of Symptoms and Risks of Child Abuse and Neglect

Subscales	Cronbach’s α	
	Test	Retest
Physical symptoms of CAN	0.756	0.753
Behavioral symptoms of CAN	0.824	0.752
Symptoms of neglect on the child	0.810	0.728
Characteristics of parents prone to abuse and neglect	0.784	0.767
Characteristics of children prone to being abused and neglected	0.229	0.332
Familial characteristics in CAN	0.612	0.662
Internal consistency	0.929	0.923

In conclusion, the analysis showed that the results are consistent, stable, and highly repeatable (Table 2).

**Table 2:** Internal consistency, reliability, and validity of the Greek version of SISRCAN (n=70) Summary of psychometric properties of the Greek version of the SISRCAN questionnaire. This includes Cronbach’s α (internal consistency), Pearson’s r (repeatability), intraclass correlation coefficient (ICC) with 95% confidence interval (CI) (test-retest reliability), and paired samples t-test statistics. Statistically significant if p<0.05 ^a^Test phase ^b^Retest phase SISRCAN: Scale for the Identification of Symptoms and Risks of Child Abuse and Neglect

Attribute	Measure	Value	Significance
Internal consistency	Cronbach’s α	0.929^a^	-
		0.923^b^	-
Repeatability	Pearson’s r	0.899	<0.001
Test-retest reliability I	ICC (95% CI)	0.947 (0.914, 0.967)	<0.001
Test-retest reliability II	Paired samples t-test	238.2 (±27.40)^a^	0.007
		242.2 (±27.42)^b^	

For the main study, Cronbach’s α coefficient, after reversing the questions with reverse meaning, was found to be equal to 0.889. This value is considered particularly high.

The total sample consisted of 493 registered midwives (479 women, 97.1%) with an average age of 44.59 (±10.33) years. More than half (282) were married (57.2%), and 312 midwives had children (63.3%). The average time lapse from the participants’ last degree was 16 years (Table 3). The average professional length of experience was 18.46 (±10.46) years. During the study period, 402 (81.5%) respondents were working in maternity hospitals, 42 (8.7%) in primary healthcare centers, 36 (7.3%) as freelancers, and 13 (2.5%) in education and other midwifery sectors. Participants provided healthcare to 11.82 (±9.98) pregnant/postpartum women/mothers per day on average.

**Table 3:** Participants’ characteristics Demographic and professional characteristics of the total sample of midwives who participated in the study (n=493)

Characteristics	Values
Sex, n (%)	
Women	479 (97.2)
Men	14 (2.8)
Age, mean (±standard deviation)	44.59 (±10.33)
Marital status, n (%)	
Single	117 (23.7)
Married	282 (57.2)
In a permanent relationship	42 (8.3)
Separated	3 (0.6)
Divorced	42 (8.5)
Having children, n (%)	
No	181 (36.7)
Yes	312 (63.3)
Number of children, n (%)	
1	71 (24.0)
2	191 (64.5)
3	30 (10.1)
4	3 (1.0)
5	1 (0.3)
Academic status, n (%)	
Bachelor’s degree from a private college	8 (1.6)
Bachelor’s degree from a public university	303 (61.5)
Master’s degree	169 (34.3)
Doctorate of philosophy	11 (2.2)
Postdoc	2 (0.4)
Time lapse from last degree in years, median (±interquartile range)	16.32 (±11.81)

Of the 493 participants surveyed, the majority (450, 91.3%) reported no personal experience of adverse childhood events (Table 4). However, 184 midwives (37.3%) indicated awareness of someone who had experienced childhood abuse. A total of 44 participants (8.9%) had previously reported suspected CAN cases, whereas 84 (17.1%) acknowledged having had suspicions but choosing not to report. The most frequently cited barriers to reporting included “feeling uncertain about the evidence” (71.4%) and “lack of trust in legal authority” (29.3%). A significant difference was found between participants with awareness of abuse and those without; participants with awareness scored higher (242.57 versus 237.26, p=0.017).

**Table 4:** Experience with child abuse Participants’ personal and professional experience with child abuse and neglect (CAN) (n=493)

Experience	Values
Have you been a victim of child abuse? n (%)	
Yes	43 (8.7)
No	450 (91.3)
Do you know someone who has been a victim of child abuse? n (%)	
Yes	184 (37.3)
No	309 (62.7)
In your work, have you ever reported a possible incident of child abuse? n (%)	
Yes	44 (8.9)
No	449 (91.1)
Did you have suspicions but chose not to report the incident? n (%)	
Yes	84 (17.1)
No	409 (82.9)

Regarding education and training, 462 respondents (94.7%) reported receiving no formal training on CAN during their pre- or post-graduation studies, 16 (3.2%) attended a program of 1-5 hours and 10 (2.0%) a program of 5-10 hours, and only five participants (1.0%) received training that lasted more than 10 hours (Table 5). In terms of continuing education, only 25 respondents (5.1%) had completed in-service training on the topic. Out of the 493 respondents, a significant proportion (344, 69.8%) felt that their in-service education was insufficient for managing CAN cases. Similarly, 355 participants (72.0%) considered their preregistration education insufficient, and only two (0.4%) rated it as absolutely sufficient to address child abuse and neglect in clinical practice.

**Table 5:** Training on CAN Formal and in-service training received by midwives on child abuse and neglect (CAN), including the duration of training programs and the perceived adequacy of their education on the subject (n=493)

Education/training on CAN	Values
Have you received formal training on CAN during your pre- or post-graduation studies? n (%)	
Yes	31 (5.3)
No	462 (94.7)
Hours of formal training on CAN, n (%)	
1-5	16 (3.2)
5-10	10 (2.0)
10+	5 (1.0)
Have you received in-service education? n (%)	
Yes	25 (5.1)
No	468 (94.9)
Was your in-service education sufficient for managing CAN cases? n (%)	
Absolutely sufficient	2 (0.4)
Very sufficient	2 (0.4)
Sufficiently sufficient	13 (2.6)
Slightly sufficient	132 (26.8)
Absolutely insufficient	344 (69.8)
Was your preregistration education sufficient for managing CAN cases? n (%)	
Absolutely sufficient	2 (0.4)
Very sufficient	1 (0.2)
Sufficiently sufficient	10 (2.0)
Slightly sufficient	125 (25.4)
Absolutely insufficient	355 (72.0)

Participants obtained an average SISRCAN score of 239.23 (standard deviation {SD}=23.60) (Table 6). Considering that the total possible score ranges from 67 to 335, this result reflects a moderate level of participants’ knowledge about the risk factors and identification of CAN. The subscale evaluating the knowledge of child characteristics linked to increased risk for child abuse and neglect (CAN) yielded the lowest mean scores, whereas the subscale titled “symptoms of neglect on the child” demonstrated the highest average score across all assessed domains.

**Table 6:** Descriptive statistics of SISRCAN Mean scores, standard deviations (SD), and theoretical score ranges for each subscale and the total score of the SISRCAN questionnaire CAN, child abuse and neglect; SISRCAN, Scale for the Identification of Symptoms and Risks of Child Abuse and Neglect

Subscales	Mean	SD	Range
Physical symptoms of CAN	72.28	7.33	19-95
Behavioral symptoms of CAN	55.82	6.56	15-75
Symptoms of neglect on the child	26.80	4.56	7-35
Characteristics of parents prone to abuse and neglect	38.43	5.96	12-60
Characteristics of children prone to being abused and neglected	19.33	3.25	6-30
Familial characteristics in child abuse and neglect	26.98	3.99	8-40
Total score	239.23	23.60	67-335

## Discussion

While the contribution of midwives to the protection of children is undeniable, little is known about the level of their knowledge internationally and at a national level. For this purpose, the Greek version of SISRCAN was translated and validated. The outcomes confirmed that the Greek SISRCAN can be used by midwives since the psychometric results showed consistency, stability, and high repeatability of the tool. After validating the questionnaire, we used it to assess the level of registered midwives’ knowledge of signs and risk factors of CAN.

Our study of 493 midwives in Greece showed that 8.1% of them have personally experienced child abuse in the past, while 37.3% indicated knowing someone who had been a victim. Although these figures are concerning, they appear relatively lower when compared to previous studies. For instance, Bozkurt et al. found that 40.6% of midwifery students reported being victims of domestic violence, and 36.2% had witnessed such violence [22]. Similarly, Elarousy et al. reported that 20.6% of nursing students had experienced child abuse [23]. In the study conducted by Buyuk, 40% of midwifery students stated that they had personally experienced abuse during childhood [12].

A significant finding of our study pertains to the limited training Greek midwives receive regarding CAN. Notably, 93.7% of participants (462 out of 493) reported receiving no formal education on CAN during their midwifery training. Additionally, 72% (n=354) considered their overall training inadequate to address CAN cases. Moreover, 95.8% (472 of 493) reported receiving minimal or no in-service training on the subject. These findings align with previous international research. For instance, Feng and Wu [24] and Salami and Alhalal [25] highlighted similar deficits in Taiwan and Saudi Arabia, respectively. Lee et al. noted that 84.9% of Taiwanese nurses had not taken any CAN-related coursework during their undergraduate studies, and 80.7% had not received in-service training, emphasizing the global need for improved education on identifying and managing suspected CAN cases [26]. Consistent with these findings, Saifan et al. reported that none of the 100 participating nurses had received training on CAN, and most were unsure how to respond effectively to suspected abuse [27]. Furthermore, a more recent study by Konukbay et al. indicated that only 29.9% of nursing students had received any training related to CAN [18].

Our study revealed that participating midwives demonstrated a moderate level of knowledge (mean score=239.23/335), a result that concurs with similar studies in the field. For example, Cevik Durmaz et al. [8] reported a mean knowledge score of 226.46 among health professionals, while Tuğut and Daşlı [7] found an average of 238.88. Previous studies of nursing students have shown scores from 237.85 to 245.22 [6,13,15]. Similarly, Sezici et al. [16] reported a mean score of 244 among midwifery students, while Buyuk [12] recorded a slightly lower average of 237.77. Other studies also corroborate these findings: Ozcevik Subasi et al. [14] reported a mean score of 264.65 among nursing students, Konukbay et al. [18] 255.27, Özbey et al. [11] 247.9, and Özkan and Karakas [9] 231.15. In Kabakoğlu and Tambağ’s study, midwives and nurses working in family health centers scored 238.2, showing a moderate level of knowledge about CAN [10]. Uysal’s Master’s thesis also indicated slightly higher knowledge scores among nurses (249.91) compared to midwives (243.21), though the difference was not statistically significant [4]. Üstündağ et al. [17], in a study involving 485 health undergraduate students, found average scores of 241.87 for female and 235.17 for male students, reinforcing the observation that female participants tend to demonstrate higher CAN knowledge, consistent with findings from the literature [6,9,11,15,28]. Moreover, Üstündağ et al. noted that students in physiotherapy and rehabilitation achieved the highest scores (mean=249.24), with midwifery students scoring 240.53 [17]. In a study by Solak et al., family physicians had a mean knowledge score of 261.5, underscoring the widespread need for enhanced CAN training across all health professions [5].

An additional noteworthy finding from this study is the relatively low average score on the subscale “characteristics of children prone to being abused and neglected.” This trend is consistent with previous literature, where similarly low scores were observed [6,7,10,11,16,18]. In several studies, scores on this subscale fell below baseline levels, highlighting a concerning gap in understanding [4,8,9,12,13]. However, in contrast, the subscale “familial characteristics in child abuse and neglect” recorded the lowest scores in the studies by Kartal and Bayraktar [15] and Üstündağ et al. [17], except among students in the audiology department, where the lowest score was found in the subscale on child characteristics prone to being abused and neglected. In the study by Ozcevik Subasi et al., the subscale “characteristics of parents prone to abuse and neglect” received the lowest ratings [14]. Consistent with the present findings, most literature agrees that the subscale “symptoms of neglect on the child” typically receives the highest mean score among all subscales [6-11,13-16,18].

Limitations and future directions

This research constitutes the first validation of the SISRCAN instrument among Greek midwives and represents the initial attempt to assess their knowledge regarding the symptoms and risk factors associated with CAN. Nonetheless, the study is subject to several limitations. Notably, the use of a convenience sampling method may limit the generalizability of the findings, as the majority of participants were based in Athens. This geographic concentration reflects partly the disproportionately large share of Greece’s midwifery workforce based in the capital. Furthermore, since the questionnaire was self-administered and covered sensitive topics, some responses may have been biased.

Currently in Greece, there is no centralized national registry for midwives, making it difficult to compile an accurate, up-to-date count of practitioners across all sectors. Current figures indicate about 0.27 midwives per 1,000 inhabitants. Given a total population in Greece of roughly 10.7 million, this corresponds to approximately 2,890 active midwives nationwide [29]. Therefore, our sample of 493 represents about 16% of the entire midwifery workforce, a notably robust proportion. Furthermore, our sample was predominantly composed of female midwives working in hospital settings. Only 14 male midwives participated, representing 2.8% of the total sample. However, this distribution likely mirrors the broader employment landscape in Greece, where over 98% of births occur within hospital environments and male midwives constitute approximately 2.9% of the registered workforce [30].

Future research should aim to include larger and more demographically diverse samples from across the country. Additionally, to strengthen the internal consistency of the SISRCAN tool, future applications should consider employing a more extensive sample size and reevaluating the inclusion of items with low factor loadings to improve the coherence and reliability of the subscales.

## Conclusions

Child abuse and neglect (CAN) remain pervasive global issues, linked to both immediate and long-term harm to children’s physical and psychological well-being. Midwives hold a critical role in the prevention, early identification, and management of CAN, underscoring their importance in safeguarding children. This study supports the Greek adaptation of the SISRCAN instrument as a valid and reliable tool for evaluating midwives’ knowledge of the risk factors and indicators associated with child maltreatment. Our findings indicate that Greek midwives possess a moderate level of knowledge regarding CAN, aligning with trends observed in international research. While there is a general understanding of key concepts, notable gaps exist, particularly in recognizing factors that increase a child’s vulnerability to abuse. These include issues closely tied to midwifery care, such as prematurity, unintended pregnancies, early infancy, and complex neonatal health needs. The limited awareness in these areas suggests a shortfall in both undergraduate and postgraduate training.

Barriers to effective child protection extend beyond knowledge deficits. Midwives also report fears of legal consequences and confusion stemming from unclear legal frameworks. These challenges hinder their ability to act confidently in suspected cases of abuse. This study offers a reliable tool for assessing midwives’ knowledge and provides valuable data to inform targeted educational programs and policy development. Its ultimate aim is to strengthen the early prevention and detection of CAN, contributing meaningfully to child protection efforts. To address these challenges, it is essential for health policy makers and academic institutions to prioritize the integration of comprehensive CAN education into midwifery curricula. Equipping midwives with the necessary knowledge and skills will enhance their capacity to advocate for and protect the health and safety of both women and children.
